# Developing a machine-learning model for real-time prediction of successful extubation in mechanically ventilated patients using time-series ventilator-derived parameters

**DOI:** 10.3389/fmed.2023.1167445

**Published:** 2023-05-09

**Authors:** Kuo-Yang Huang, Ying-Lin Hsu, Huang-Chi Chen, Ming-Hwarng Horng, Che-Liang Chung, Ching-Hsiung Lin, Jia-Lang Xu, Ming-Hon Hou

**Affiliations:** ^1^Division of Chest Medicine, Department of Internal Medicine, Changhua Christian Hospital, Changhua, Taiwan; ^2^Artificial Intelligence Development Center, Changhua Christian Hospital, Changhua, Taiwan; ^3^Institute of Genomics and Bioinformatics, National Chung Hsing University, Taichung, Taiwan; ^4^Ph.D. Program in Medical Biotechnology, National Chung Hsing University, Taichung, Taiwan; ^5^Department of Applied Mathematics, Institute of Statistics, National Chung Hsing University, Taichung, Taiwan; ^6^Division of Chest Medicine, Department of Internal Medicine, Yuanlin Christian Hospital, Changhua, Taiwan; ^7^Department of Recreation and Holistic Wellness, MingDao University, Changhua, Taiwan; ^8^Graduate Institute of Biotechnology, National Chung Hsing University, Taichung, Taiwan; ^9^Department of Life Sciences, National Chung Hsing University, Taichung, Taiwan

**Keywords:** extubation, intensive care unit, machine learning, mechanical ventilation, prediction model

## Abstract

**Background:**

Successful weaning from mechanical ventilation is important for patients admitted to intensive care units. However, models for predicting real-time weaning outcomes remain inadequate. Therefore, this study aimed to develop a machine-learning model for predicting successful extubation only using time-series ventilator-derived parameters with good accuracy.

**Methods:**

Patients with mechanical ventilation admitted to the Yuanlin Christian Hospital in Taiwan between August 2015 and November 2020 were retrospectively included. A dataset with ventilator-derived parameters was obtained before extubation. Recursive feature elimination was applied to select the most important features. Machine-learning models of logistic regression, random forest (RF), and support vector machine were adopted to predict extubation outcomes. In addition, the synthetic minority oversampling technique (SMOTE) was employed to address the data imbalance problem. The area under the receiver operating characteristic (AUC), F1 score, and accuracy, along with the 10-fold cross-validation, were used to evaluate prediction performance.

**Results:**

In this study, 233 patients were included, of whom 28 (12.0%) failed extubation. The six ventilatory variables per 180 s dataset had optimal feature importance. RF exhibited better performance than the others, with an AUC value of 0.976 (95% confidence interval [CI], 0.975–0.976), accuracy of 94.0% (95% CI, 93.8–94.3%), and an F1 score of 95.8% (95% CI, 95.7–96.0%). The difference in performance between the RF and the original and SMOTE datasets was small.

**Conclusion:**

The RF model demonstrated a good performance in predicting successful extubation in mechanically ventilated patients. This algorithm made a precise real-time extubation outcome prediction for patients at different time points.

## Introduction

Although invasive mechanical ventilation is an important part of critical care, prolonged mechanical ventilation results in ventilator-associated complications, morbidity, mortality, and increased hospitalization costs (1, 2). As soon as the initiating factors causing respiratory failure begin to improve, weaning and extubation are essential; thus, the ability to determine whether a patient is ready for extubation is crucial (3). However, extubation is sometimes unsuccessful, and approximately 5.2–20% of cases require reintubation (4–6). Extubation failure has consequences such as the need for a tracheotomy, the occurrence of pneumonia, and pulmonary damage induced by mechanical ventilation (7).

Discontinuation of ventilatory support can be challenging for physicians, mostly because the pathophysiology of weaning failure is complex and not fully understood. Clinicians can predict failed extubation with a sensitivity and specificity of 57 and 31%, respectively (8). Currently, the rapid shallow breathing index (RSBI; f/Vt) is the most commonly used predictor of ventilator weaning (8). However, the pooled sensitivity and specificity of an RSBI of less than 105 breaths/min/L in predicting extubation success are 83 and 58%, respectively. This suggests that the RSBI has only a moderate ability to rule out extubation success and does not sufficiently predict successful extubation (9). Furthermore, numerous parameters have been reported as predictors of weaning outcomes, including cough strength, duration of mechanical ventilation, and diaphragmatic function (10–12). However, none of these clinical parameters have a better, real-time, and comprehensive ability to predict extubation outcomes.

Outcome prediction methods using machine-learning models have recently been developed in many areas of healthcare research (13–15). Traditional statistical methods mainly focus on relationships between variables. In contrast, machine learning can be applied to big data using different approaches and can make more accurate predictions (16). Recently, some studies have explored the ability of machine-learning models to accurately predict extubation outcomes (17–22). Most studies used demographic data (e.g., age, sex, duration of mechanical ventilation, and reasons for respiratory failure), physiological data (e.g., the Acute Physiology and Chronic Health Evaluation [APACHE] II score, respiratory rate [RR], blood pressure, heart rate, peripheral oxygen saturation, and temperature), and laboratory data (e.g., lymphocyte, hematocrit, glucose, and sodium levels) (17–22). Because these studies collected raw data during a certain period (i.e., 2 h (21), 4 h (20) or longer), their models perform only static and not dynamic predictions. In addition, these studies used numerous features [ranging from 8 to 78 features (23)] that are difficult to gather in clinical practice in their algorithms, which makes their widespread application in hospitals difficult and reduces their accessibility.

Therefore, this study aimed to develop a machine-learning model with good accuracy to predict successful extubation only using the time-series parameters obtained from mechanical ventilators that are measured every second. The model performed a time-phased and immediate prediction within minutes to hours before extubation and required no integration of data from multiple systems via an electronic health record (EHR) or similar. Additionally, the trend change in extubation outcome prediction for patients would be provided to clinicians in future studies.

## Methods

### Study participants

This study was conducted in the mixed medical–surgical intensive care unit (ICU) of Yuanlin Christian Hospital, a 250 bed local community hospital with 20 ICU beds for adults, from August 1, 2015 to November 30, 2020. The inclusion criteria were as follows: (1) patients aged ≥20 years, (2) those diagnosed with acute respiratory failure who received invasive mechanical ventilation for more than 24 h consecutively, and (3) those who underwent extubation during hospitalization in the ICU. The exclusion criteria were as follows: (1) patients who received mechanical ventilation with a tracheostomy or nasal endotracheal tube, (2) those who had undergone accidental extubation or self-extubation, and (3) those who were difficult to wean from mechanical ventilation for more than 21 days and were further transferred to the Respiratory Care Center. If a patient received multiple mechanical ventilation sessions during an ICU visit, every mechanical ventilation session was collected individually. Data were retrospectively collected and analyzed. Therefore, informed consent was waived, and the study was approved by the Institutional Review Board of Changhua Christian Hospital (approval no.: 210716). All procedures were performed in accordance with the Declaration of Helsinki.

### Ventilator setup

We used Servo I (Maquet, Solna, Sweden) ventilators. All ventilator settings (i.e., the modes, expiratory tidal volume (Vte), RR, positive end-expiratory pressure (PEEP), fraction of inspired oxygen (FiO_2_), rising time, and support pressure) and measurement parameters (i.e., the Vte, RR, peak airway pressure (Ppeak), mean airway pressure (Pmean), PEEP, and FiO_2_) were recorded from the patients every second through a data port on the back of the ventilator and were stored in the database. Respiratory therapists chose one data point every hour to prepare the medical records in the hospital information system.

### Weaning procedure

The weaning procedure followed the protocol established on the basis of recommendations of the literature and medical guidelines (24). Sedatives, hypnotics, and narcotics were discontinued at least 8 h before weaning. The patients were identified as ready for weaning based on clinical stability criteria. The weaning process was initiated in the pressure-support ventilation (PSV) mode, followed by a spontaneous breathing trial (SBT) in a T-piece for 30 min. The RSBI was calculated for patients who passed the SBT. The cuff leak test was performed before extubation.

### Data collection

We collected patients’ clinical characteristics (i.e., demographics, the APACHE II score, reasons for intubation, and length of hospital stay) and weaning profiles from their medical records. The patients’ ventilation parameters (i.e., Vte, RR, Ppeak, Pmean, PEEP, and FiO_2_) were obtained from the ventilator as a time-series dataset in seconds before extubation. Extubation failure was defined as the need for reintubation within 48 h after extubation. Post-extubation respiratory failure was defined as the presence of two or more of the following parameters: RR of more than 35 min; arterial oxyhemoglobin saturation of less than 88% despite adequate additional oxygen; pH of less than 7.20, which was decreased from the onset; hypercapnia with PaCO_2_ of more than 45 mmHg; a decreased level of consciousness; and abdominal paradox (25). In the extubation success cohort, 4 h time-series data before extubation were collected. Because of imbalanced datasets between the extubation success and failure cohorts, we hypothesized that early extubation would fail in the extubation failure cohort. However, this was not certain in the extubation success cohort. Therefore, we used 12 h time-series data obtained from the ventilator before extubation in the extubation failure cohort and evenly divided it into 4 h segments. Using this process, we obtained a three-fold expansion in the extubation failure cohort (Supplementary Figure S1).

### Data processing

For data processing, we first used the averaging method to separate the patients’ ventilation parameters per 1, 30, 60, 120, 180, and 300 s. Subsequently, we squared and calculated the degree of difference in the ventilation parameters to increase the dimensions.

### Feature extraction

For feature extraction, we used the information gain method to calculate the entropy of the time-series dataset of ventilation parameters for extubation success or failure (26). The entropy value is expressed between 0 and 1, where 0 indicates that the field cannot be divided into extubation success or failure and 1 indicates that the field can be effectively divided. We obtained the raw data and calculated the degree of difference in the ventilation parameters to calculate the feature importance (FI) in the 1-, 30-, 60-, 120-, 180-, and 300 s datasets from the information gain algorithm results. Recursive feature elimination (RFE) was applied to select the most important features. It uses a classifier to rank the features and recursively removes the weakest features (27). The process of removing the weakest features continues until the required number of features is reached.

### Modeling

**Figure 1:**
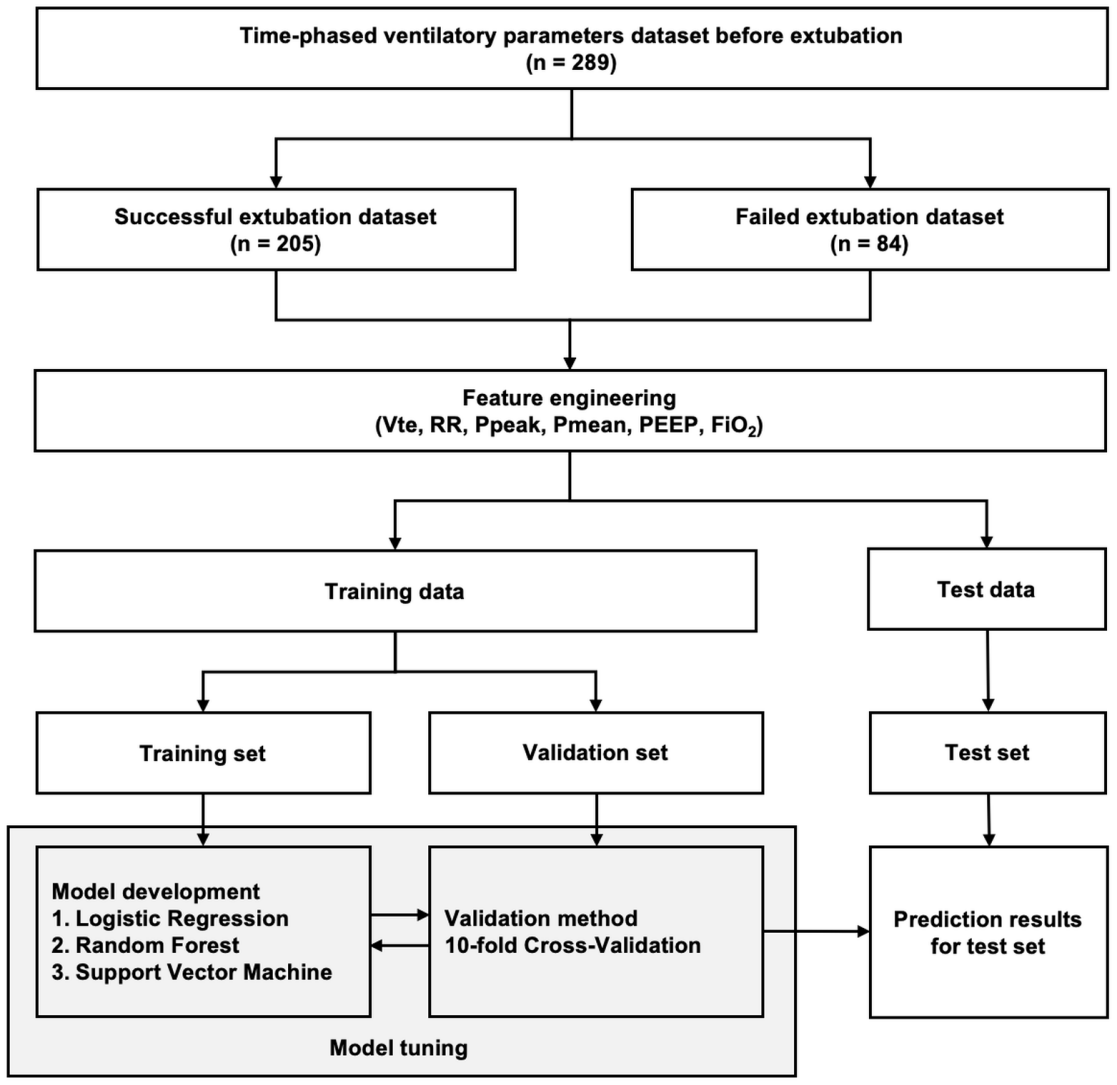
Architecture of the machine-learning model for predicting extubation outcomes.

The ventilator-derived parameter time-series dataset was divided into two parts: testing (30 min before extubation) and training (the remaining 210 min) (Figure 1). Python (version 3.7) was used for all analyses. In the training stage, we used 10-fold cross-validation to train the model. The 10-fold cross-validation was divided into 10 datasets: one dataset was used as a validation set, and the remaining datasets were used as training sets. To develop the predictive models, we used the following supervised machine-learning methods, which are the most prominent and up-to-date methods for classification problems: logistic regression, random forest (RF), and support vector machine (SVM) (28–30). The logistic regression model accurately predicts the probability of the binary-dependent variable using maximum likelihood estimation to produce the regression coefficient. The decision tree approach is a tree-like model of decisions that can mathematically forecast the optimum option. A simple decision tree was created using an optimized version of the classification and regression tree algorithm (31). To determine the split point, we used the Gini index as a statistic. The Gini index measures the likelihood of erroneously classifying a dataset at random. Because the drawbacks of the simple decision tree are instability and the possibility of overfitting, the RF was used to improve the prediction performance (31). The RF is an ensemble classifier that uses majority voting to aggregate many decision trees. The SVM, a method for defining a high-dimensional boundary that clearly classifies data points, was also used. To address data imbalance, the synthetic minority oversampling technique (SMOTE), the method endorsed in the literature, was used (32). We evaluated the prediction performance using the area under the receiver operating characteristic curve (AUC), sensitivity, specificity, positive predictive value, negative predictive value, F1 score, and accuracy, along with the 10-fold cross-validation. The outcome variable was binary, with 1 indicating successful weaning, and 0 indicating failed weaning. The predictive value of the machine learning models was between 0 and 1. A value closer to 1 indicated a greater probability of successful extubation, and a value closer to 0 indicated a greater probability of extubation failure.

### Statistical analyses

Continuous variables are expressed as medians and interquartile ranges (IQRs; the differences between the 25th and 75th percentiles), and categorical variables are presented as percentages. The chi-square test and Mann–Whitney U test were used to detect differences in the categorical and continuous variables. Differences with *p*-values of less than 0.05 were considered statistically significant. To interpret the model better, we employed the SHapley Additive exPlanations (SHAP) method to provide consistent and locally accurate attribution values for each feature within the prediction model (33). The probability density distribution was obtained to better discriminate between the extubation success and failure cohorts. All statistical analyses were performed using Python and MedCalc (version 20.027; MedCalc, Mariakerke, Belgium).

## Results

**Figure 2:**
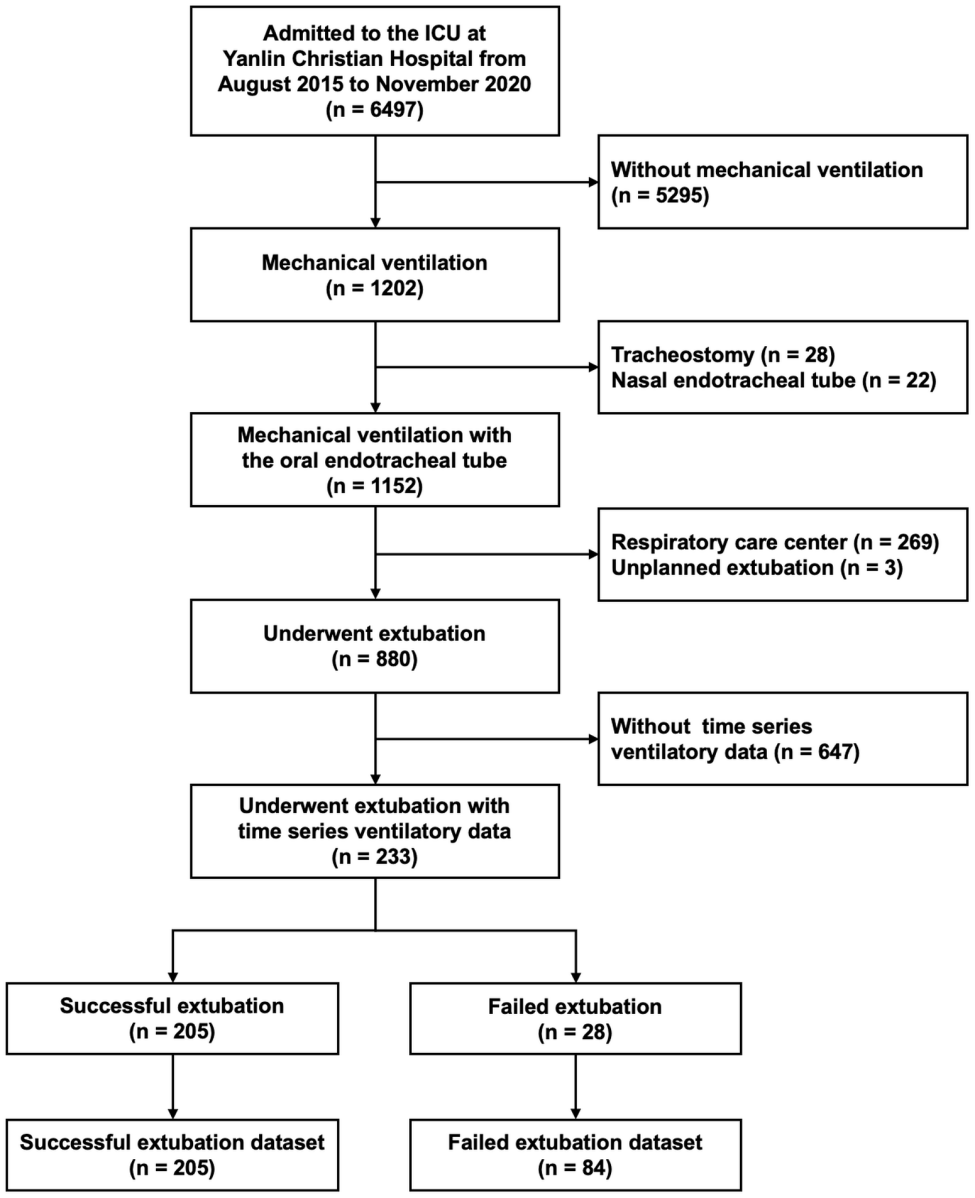
Flowchart of study participant enrollment.

In this study, 233 patients met the inclusion criteria, of whom 28 (12.0%) failed extubation (Figure 2). Demographic, clinical, and weaning profiles are shown in Table 1. The APACHE II scores and diagnoses at ICU admission were comparable between the extubation success and failure cohorts. The weaning profiles, including maximal inspiratory and expiratory pressures and mean RSBI values were not statistically different between the two cohorts. The duration of hospital stay was significantly longer in the extubation failure cohort than in the extubation success cohort (median: 18.3 vs. 14.7 days; *p* = 0.006).

**Table 1:** Demographic characteristics and weaning profiles of the participants.

Characteristic	All (*n* = 233)		Success (*n* = 205)		Failure (*n* = 28)		*p*
Age, years	73.0	(61.8–81.3)	73.0	(61.0–82.0)	72.5	(64.5–80.5)	0.980
Gender (%)							
Male	147	(63.1)	132	(64.4)	15	(53.6)	0.267
Female	86	(36.9)	73	(35.6)	13	(46.4)	
APACHE II score	21.0	(16.0–26.0)	21.0	(16.0–26.0)	20.0	(16.0–25.5)	0.982
Hospital stay, days	15.2	(10.1–22.3)	14.7	(9.7–21.7)	18.3	(14.3–25.4)	0.006
Mechanical ventilation duration, days	3.8	(2.3–7.1)	3.8	(2.2–7.4)	5.4	(3.0–7.0)	0.311
Reasons for intubation (%)							
Pneumonia	106	(45.5)	94	(45.9)	12	(42.9)	0.369
ICH	28	(12.0)	24	(11.7)	4	(14.3)	
Post surgery	25	(10.7)	23	(11.2)	2	(7.1)	
AMI	19	(8.2)	18	(8.8)	1	(3.6)	
Septic shock	19	(8.2)	18	(8.8)	1	(3.6)	
Medical emergency	19	(8.2)	16	(7.8)	23	(10.7)	
Stroke	9	(3.9)	6	(2.9)	3	(10.7)	
Others	8	(3.4)	6	(2.9)	3	(7.1)	
Weaning profiles							
Pimax	24.0	(−32.0–40.0)	24.0	(−32.0–40.0)	−6.0	(−40.0–40.0)	0.281
Pemax	44.0	(32.0–60.0)	45.0	(34.0–60.0)	41.0	(24.0–60.0)	0.353
RSBI_average	56.2	(37.9–79.3)	57.4	(37.4–86.4)	50.0	(41.4–69.0)	0.543
RSBI_pass (%)	200	(85.8)	173	(84.4)	27	(96.4)	0.087
Cuff leak test_average	346	(160–500)	350	(162–500)	265	(139–476)	0.574
Cuff leak test_pass (%)	198	(85.0)	175	(85.4)	23	(82.1)	0.655

AMI, acute myocardial infarction; APACHE II score, acute physiology and chronic health evaluation II score; ICH, intracerebral hemorrhage; Pemax, maximal expiratory pressure; Pimax, maximal inspiratory pressure; RSBI, rapid shallow breathing index.

The time-series dataset of the ventilation parameters is presented in Supplementary Table S1. The extubation failure cohort was more likely to have a lower Vte and higher Ppeak and Pmean than the extubation success cohort. The average IQR of PEEP and FiO_2_ decreased in the extubation success cohort. The mean RR values were not statistically different between the two cohorts. No statistically significant differences were observed in the ventilator-derived parameters between the training and testing datasets.

**Figure 3:**
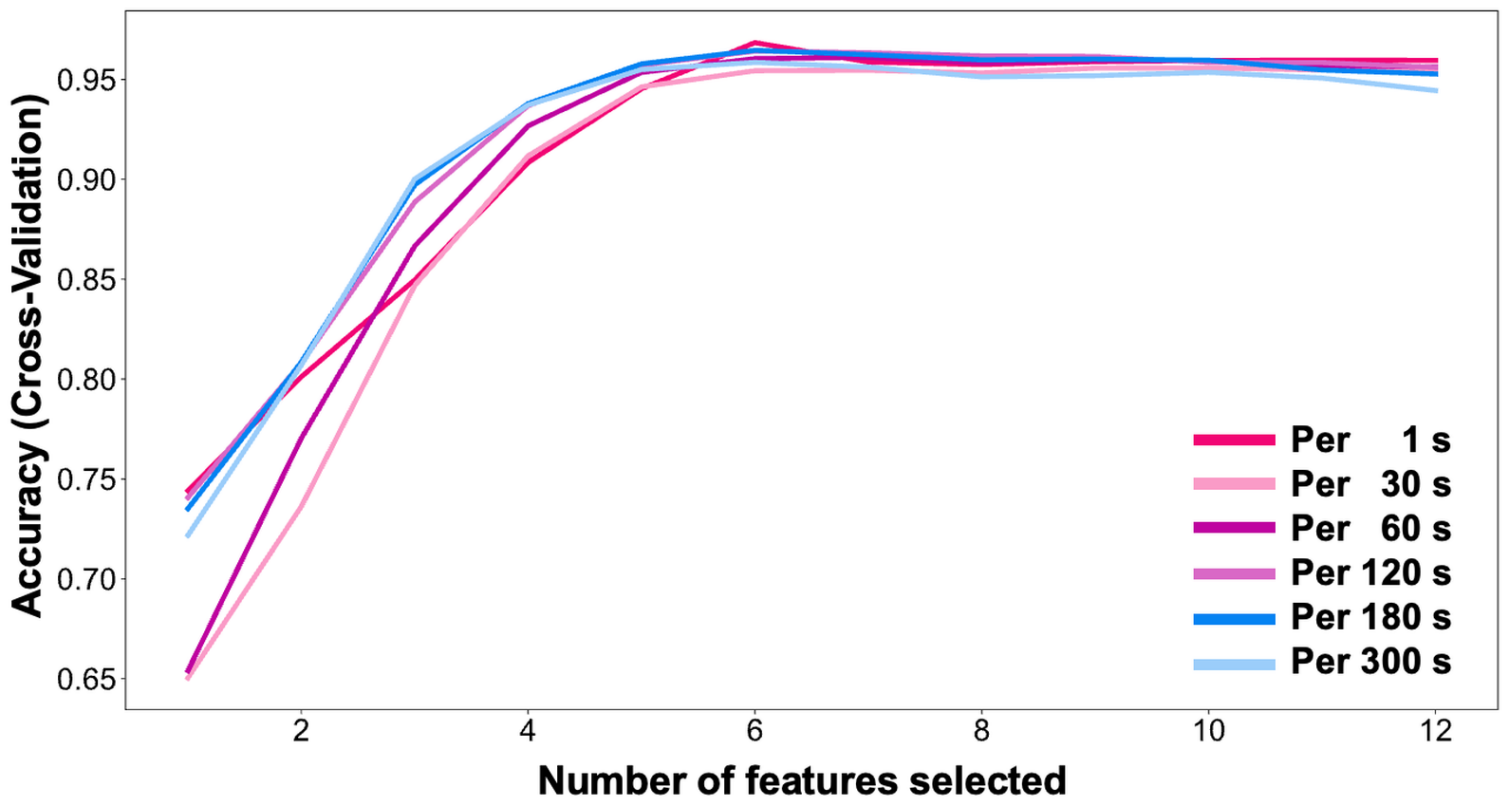
Feature selection using recursive feature elimination (RFE).

Feature selection algorithms were applied to the training dataset containing the 12 variables (Figure 3). Based on the output of the feature selection algorithms and literature review, the accuracy plateaued after selecting six variables in the 1-, 30-, 60-, 120-, 180-, and 300 s datasets. The ventilatory variables had the highest importance of characteristics per 180 s. The six most important variables contributing to the extubation outcomes were FiO_2_ (FI: 0.144) and Ppeak (FI: 0.142), PEEP (FI: 0.105), Pmean (FI: 0.066), RR (FI: 0.027), and Vte (FI: 0.025), in descending order (Table 2). The predictive effect was poor when the degree of difference was considered. We used the raw data of the six ventilator-derived parameters as the input variables.

**Table 2:** Feature importance of time-series ventilator-derived parameters with the mean of different intervals of seconds.

Ventilatory parameters	Per 1 s	Per 30 s	Per 60 s	Per 120 s	Per 180 s	Per 300 s
FiO_2_	0.108	0.127	0.133	0.140	0.144	0.128
Ppeak	0.083	0.121	0.131	0.139	0.142	0.140
PEEP	0.043	0.076	0.088	0.099	0.105	0.113
Pmean	0.034	0.057	0.066	0.071	0.066	0.065
RR	0.022	0.025	0.026	0.027	0.027	0.031
Vte	0.018	0.024	0.024	0.025	0.025	0.027
FiO_2__DOD	0	0	0.002	0.002	0.004	0.005
Ppeak_DOD	0	0.004	0.038	0.002	0.003	0.004
PEEP_DOD	0	0.003	0.004	0	0.006	0
Pmean_DOD	0	0.004	0.003	0	0	0
RR_DOD	0.001	0.009	0.007	0.008	0.005	0.005
Vte_DOD	0.001	0.007	0.006	0.006	0.008	0.010

DOD, degree of difference; FiO_2_, fraction of inspiration oxygen; PEEP, positive end-expiratory pressure; Pmean, mean airway pressure; Ppeak, peak airway pressure; RR, respiratory rate; sq, squared; Vte, expiratory tidal volume.

We used the machine-learning methods of logistic regression, RF with the original and SMOTE datasets, and SVM with all the variables per 180 s as input variables to predict extubation outcomes. To determine the optimal model, a grid search with the 10-fold cross-validation for hyperparameter tuning (Supplementary Table S2) for each algorithm was conducted. The prediction performances of the four machine learning models are listed in Table 3. RF with the original dataset exhibited an AUC of 0.976 (95% confidence interval [CI], 0.975–0.976), accuracy of 94.0% (95% CI, 93.8–94.3%), and F1 score of 95.8% (95% CI, 95.7–96.0%). Similarly, the RF with the SMOTE dataset showed an AUC of 0.979 (95% CI, 0.978–0.980), accuracy of 92.9% (95% CI, 92.7–93.1%), and F1 score of 94.9% (95% CI, 94.7–95.0%), indicating that the RF model performs better than the logistic regression and SVM models. Furthermore, the difference in the performance between the RF with the original and SMOTE datasets was small. This implies that the effect of the data imbalance is less important in our dataset.

**Table 3:** Performance comparison among different machine-learning models.

Model		AUC (95% CI)	Sensitivity (%) (95% CI)	Specificity (%) (95% CI)	PPV (%) (95% CI)	NPV (%) (95% CI)	F1 score (%) (95% CI)	Accuracy (%) (95% CI)
Logistic regression	original	0.730 (0.729–0.730)	14.7 (14.3–15.0)	96.6 (96.5–96.6)	63.7 (63.2–64.2)	73.4 (73.3–73.5)	83.4 (83.4–83.5)	72.8 (72.7–72.9)
	SMOTE	0.728 (0.727–0.728)	64.1 (63.7–64.4)	68.5 (68.1–68.9)	45.5 (45.2–45.7)	82.3 (82.2–82.4)	74.8 (74.5–75.0)	67.2 (67.0–67.4)
Random forest	original	0.976 (0.975–0.976)	87.5 (87.0–87.9)	96.7 (96.5–96.9)	91.6 (91.1–92.1)	95.0 (94.8–95.1)	95.8 (95.7–96.0)	94.0 (93.8–94.3)
	SMOTE	0.979 (0.978–0.980)	92.5 (92.1–92.9)	93.0 (92.7–93.3)	84.4 (83.9–85.0)	96.8 (96.6–97.0)	94.9 (94.7–95.0)	92.9 (92.7–93.1)
Support vector machine	original	0.860 (0.858–0.861)	51.3 (51.0–51.6)	97.3 (97.2–97.4)	88.5 (88.2–88.8)	83.0 (82.9–83.1)	89.6 (89.5–89.6)	83.9 (83.8–84.0)
	SMOTE	0.871 (0.870–0.872)	63.3 (62.9–63.7)	91.4 (91.3–91.6)	75.2 (74.9–75.5)	85.9 (85.8–86.0)	88.6 (88.5–88.7)	83.3 (83.1–83.4)

AUC, area under receiver operating characteristic; CI, confidence interval; NPV, negative predictive value; PPV, positive predictive value.

The performance of the RF model with the training, validation, and testing datasets is presented in Supplementary Table S3. There was no obvious decrease in the AUC and accuracy of the RF model from the training to validation or testing datasets. In other words, the overfitting issue was limited in our RF model.

To identify the features that had the most influence on the prediction model, we constructed the SHAP summary plot of RF (Supplementary Figure S2A), which depicts how the high and low feature values relate to SHAP values in the training dataset. According to the prediction model, the higher the SHAP value of a feature, the more likely the extubation is successful. The most important predictive feature of successful extubation was the Ppeak value before extubation. For ventilatory characteristics, lower Ppeak and Pmean and higher Vte and RR in the last 30 min were predictive of extubation success. Supplementary Figure S2B shows an example of successful extubation with the SHAP value prediction result. With higher Ppeak and Vte, the output value of the probability of successful extubation was greater than 0.95. However, with lower Vte, Pmean, and Ppeak in the extubation failure cohort, the RF module presumed that the probability of successful extubation was 0 (Supplementary Figure S2C).

The distribution of the probability density of the RF predictive values is provided in Supplementary Figure S3. The predictive value of the successful extubation cohort using the RF model was higher than 0.7. In contrast, the predictive value of the extubation failure cohort was lower than 0.7. Therefore, the best cuff-off value to distinguish the prediction of the weaning outcome of the RF model was 0.7.

## Discussion

In this study, we developed a machine-learning model using only ventilator-derived data for real-time prediction of successful extubation of mechanically ventilated patients in an ICU. The RF model demonstrated good prediction performance, with an AUC of 0.976 (95% CI, 0.975–0.976) and accuracy rate of 94.0% (95% CI, 93.8–94.3%). Considering that the input ventilatory variables are per 180 s, this model can predict extubation outcomes for a single patient every 3 min. As the model uses only six ventilator-driven parameters, it can be easily applied to improve intensivists’ clinical decision-making.

In this study, we selected the most common algorithms to use in machine learning applications, including logistic regression, RF, and SVM. Logistic regression was prioritized due to its high computational efficiency, fast prediction speed, and low storage space requirements. On the other hand, RF was an ensemble learning method that generates multiple tree structures to learn from features and uses a voting mechanism for the final prediction. SVM had an advantage in processing high-dimensional data and generating hyperplane decisions from a portion of the data. Our study found that logistic regression and SVM suffered from overfitting issues during model training. These algorithms were primarily designed to find the best hyperplane, but the training data used in our study had little significant difference between the parameters of success and failure results. In contrast, the tree structure of RF allowed for exploration of different paths and parameters. However, the disadvantage of the RF model was that the tree structure might become too large to make the prediction results difficult to explain.

This study is unique in that it used a time-series dataset that included patients’ ventilatory parameters measured every second. Hagen et al. utilized a dataset of tidal volume measurements to develop a personalized clinical prediction model capable of predicting tidal volume behavior and providing alerts with 10% accuracy 1 hour ahead (34). The primary objective of their model was to prevent the occurrence of lung injury, rather than being used for extubation prediction. A recent study also used electronic medical records and ventilator variables to develop a machine-learning model to predict the successful weaning of patients in respiratory care centers (22). They used 26 feature variables and seven models—logistic regression, RF, SVM, *k*-nearest neighbors, extreme gradient boosting (XGBoost), light gradient boosting machine, and multilayer perceptron—to establish the prediction model. The XGBoost algorithm had the best performance in their study, with an AUC value of 0.868 and accuracy of 85.1%. However, the model could only provide prediction results every 24 h. Another study used historical ICU data extracted from MIMIC-III with a convolutional neural network to predict extubation readiness within the next hour for a given patient (35). However, the algorithm, which was developed by Jia et al., still used 25 variables for an AUC and accuracy of 0.94 and 86%, respectively. Our study used only six time-series ventilator-derived variables to developed an RF model with an AUC of 0.976 (95% CI, 0.975–0.976) and accuracy of 94.0% (95% CI, 93.8–94.3%) that could dynamically provide the prediction result every 3 min. Thus, it can easily be employed in a clinical setting to improve treatment decision making by critical-care physicians.

Patterns of spontaneous breathing in mechanically ventilated patients are potential markers of weaning outcomes. Variability in breathing during weaning from mechanical ventilation may be useful in clinical decision making (36). In recent studies, low respiratory variability was associated with weaning failure during mechanical ventilation (37). According to Bien et al., patients who required noninvasive or invasive mechanical ventilation within 48 h had a considerably reduced quantitative variability of tidal volume during a 30 min SBT (38). Similarly, Sarlabous et al. developed an entropy-based technique to accurately detect patient–ventilator asynchronies (39). Correspondingly, we used a machine-learning approach with ventilatory parameters derived from a time-series dataset to predict extubation success with an accuracy of 94.0% (95% CI, 93.8–94.3%).

Although artificial intelligence (AI) technologies have made remarkable advances in various fields, the use of AI algorithms with the black-box issue in healthcare remains rare because of physicians’ tendency to act only after understanding the rationale behind the results (40). Given the potentially catastrophic consequences of a bad medical decision, particularly in critical-care medicine, the black-box issue leads physicians to distrust AI models when no rationale is provided (41). However, owing to nonlinear relationships in the data, physicians find it difficult to interpret these multiple features simultaneously. We believe that this study provides a satisfactory prediction of extubation outcomes in mechanically ventilated patients as the first step. Further work is urgently needed to interpret the major features and fluctuations of the parameters that the algorithm prioritizes.

The study had several advantages, including addressing a clinically important question, utilizing machine learning techniques to develop a model using only ventilator-derived parameters, employing a robust methodology with feature selection and cross-validation, and addressing the data imbalance problem with SMOTE. Nevertheless, this study had several limitations that must be addressed. First, the definition of extubation failure remains controversial. The definition adopted in this study was reintubation within 48 h after extubation. Noninvasive ventilation or high-flow oxygen therapy with the potential to prevent reintubation were excluded. Further studies should be conducted to distinguish post-extubation management using different time intervals (e.g., 72 h after extubation). Second, PSV was the ventilator weaning mode used in this study. However, several weaning modes have been used in clinical practice. Further studies are needed on extubation failure prediction using other weaning modes. Third, because this was a single-center study, external validation is required. However, the data used in this study were routinely collected in a real-world setting. Fear of generalizability should be alleviated to a large extent.

## Conclusion

In summary, this study developed a machine-learning method to predict extubation success using only time-series ventilator-derived parameters in mechanically ventilated patients. Among the models examined in this study, the RF model had the highest accuracy. The algorithm demonstrated the capability of predicting extubation outcomes every 3 min for individual patients, while providing precise and real-time predictions of extubation outcomes at various time points. The precise predictions generated by the algorithm have the potential to mitigate complications related to mechanical ventilation and associated medical costs. More prospective studies of AI interventions in decision-making regarding mechanical-ventilator weaning are needed.

## Data availability statement

Data are available from the corresponding author on reasonable request.

## Ethics statement

The study was approved by the Institutional Review Board of Changhua Christian Hospital (approval no.: 210716). The Institutional Review Board waived the need for informed consent considering the retrospective nature of data collected.

## Author contributions

K-YH and Y-LH contributed to the data analysis. H-CC, M-HH, and C-LC contributed to the data collection. C-HL, J-LX, and M-HH contributed to data interpretation. J-LX and M-HH takes responsibility for the content of the manuscript, including the data and analysis. All authors contributed to the article and approved the submitted version.

## Funding

This research was funded by National Chung Hsing University and Changhua Christian Hospital Joint Research Program (No. NCHU-CCH 11003).

## Conflict of interest

The authors declare that the research was conducted in the absence of any commercial or financial relationships that could be construed as a potential conflict of interest.

## Publisher’s note

All claims expressed in this article are solely those of the authors and do not necessarily represent those of their affiliated organizations, or those of the publisher, the editors and the reviewers. Any product that may be evaluated in this article, or claim that may be made by its manufacturer, is not guaranteed or endorsed by the publisher.
