# Supplementary material for: Developing a machine-learning model for real-time prediction of successful extubation in mechanically ventilated patients using time-series ventilator-derived parameters
Source: Front Med (Lausanne). 2023 May 9;10:1167445. doi: 10.3389/fmed.2023.1167445 (PMC10203709; doi:10.3389/fmed.2023.1167445)
Supplement: Supplementary file 1 [file Table_1.DOCX]

**Supplementary Table 1.**

Ventilator-derived parameters of the training and testing datasets.

| Ventilatory data | All (n = 289) | | Success (n = 205) | | Failure (n = 84) | | *p* |
| --- | --- | --- | --- | --- | --- | --- | --- |
| Vte |  |  |  |  |  |  |  |
| Training data | 522.3 | (435.5–612.9) | 527.3 | (433.7–626.0) | 512.1 | (439.2–588.2) | < 0.001 |
| Testing data | 510.8 | (420.6–609.2) | 511.9 | (420.2–621.1) | 508.7 | (421.5–591.7) | 0.027 |
| RR |  |  |  |  |  |  |  |
| Training data | 16.3 | (13.0–20.3) | 16.4 | (12.7–20.4) | 16.2 | (13.6–20.0) | 0.138 |
| Testing data | 16.9 | (13.2–20.8) | 17.0 | (12.7–21.4) | 16.5 | (13.9–20.0) | 0.396 |
| Ppeak |  |  |  |  |  |  |  |
| Training data | 17.8 | (16.0–19.0) | 17.5 | (16.0–18.7) | 18.9 | (17.0–20.0) | < 0.001 |
| Testing data | 17.6 | (15.9–19.0) | 17.4 | (15.9–18.1) | 18.9 | (16.6–20.0) | < 0.001 |
| Pmean |  |  |  |  |  |  |  |
| Training data | 8.6 | (7.9–9.4) | 8.5 | (7.9–9.2) | 8.9 | (7.9–10.4) | < 0.001 |
| Testing data | 8.5 | (7.8–9.3) | 8.5 | (7.8–9.1) | 8.7 | (7.9–10.3) | < 0.001 |
| PEEP |  |  |  |  |  |  |  |
| Training data | 5.0 | (4.4–5.8) | 5.0 | (4.5–5.5) | 5.0 | (4.3–6.2) | < 0.001 |
| Testing data | 5.0 | (4.4–5.7) | 5.0 | (4.5–5.4) | 5.0 | (4.3–6.1) | < 0.001 |
| FiO_2_ |  |  |  |  |  |  |  |
| Training data | 31.0 | (30.1–31.3) | 31.0 | (30.0–31.0) | 31.0 | (30.9–35.2) | < 0.001 |
| Testing data | 31.0 | (30.2–32.0) | 31.0 | (30.1–31.3) | 31.0 | (30.9–35.3) | < 0.001 |

FiO_2_, fraction of inspiration oxygen; PEEP, positive end-expiratory pressure; Pmean, mean airway pressure; Ppeak, peak airway pressure; RR, respiratory rate; Vte, expiratory tidal volume.

Footnotes: the percentage of missing values is 0.11% in Vte, 0.17% in RR, 0.12% in Ppeak, 0.11% in Pmean, < 0.01% in PEEP and FiO_2_.
