# Supplementary material for: Developing a machine-learning model for real-time prediction of successful extubation in mechanically ventilated patients using time-series ventilator-derived parameters
Source: Front Med (Lausanne). 2023 May 9;10:1167445. doi: 10.3389/fmed.2023.1167445 (PMC10203709; doi:10.3389/fmed.2023.1167445)
Supplement: Supplementary file 2 [file Table_2.DOCX]

**Supplementary Table 2.**

Hyper-parameter range for experiments.

| Method | Hyper-Parameter | Values |
| --- | --- | --- |
| Random Forest | n_estimators | 100 |
|  | criterion | gini |
|  | max_depth | None |
|  | min_samples_split | 2 |
| Logistic Regression | penalty | L2 |
|  | tol | 0.0001 |
|  | fit_intercept | True |
|  | max_iter | 100 |
|  | solove | lbfgs |
| Support Vector Machine | penalty | L2 |
|  | loss | squared_hinge |
|  | tol | 0.0001 |
|  | max_iter | 1000 |
