# Supplementary material for: Developing a machine-learning model for real-time prediction of successful extubation in mechanically ventilated patients using time-series ventilator-derived parameters
Source: Front Med (Lausanne). 2023 May 9;10:1167445. doi: 10.3389/fmed.2023.1167445 (PMC10203709; doi:10.3389/fmed.2023.1167445)
Supplement: Supplementary file 3 [file Table_3.DOCX]

**Supplementary Table 3.**

Performance comparison of the random forest (RF) model with training, validation, and testing datasets.

| Model | AUC  (95% CI) | Sensitivity (%)  (95% CI) | Specificity (%)  (95% CI) | PPV (%)  (95% CI) | NPV (%)  (95% CI) | F1 score (%)  (95% CI) | Accuracy (%)  (95% CI) |
| --- | --- | --- | --- | --- | --- | --- | --- |
| Random Forest original |  |  |  |  |  |  |  |
| Training | 1.000  (1.000 – 1.000) | 99.8  (99.8 – 99.8) | 99.9  (99.9 – 99.9) | 99.8  (99.8 – 99.9) | 99.9  (99.9 – 99.9) | 99.9  (99.9 – 99.9) | 99.9  (99.9 – 99.9) |
| Validation | 0.990  (0.989 – 0.992) | 92.7  (92.1 – 93.3) | 98.1  (97.9 – 98.3) | 95.2  (94.8 – 95.6) | 97.0  (96.8 – 97.3) | 97.6  (97.4 – 97.7) | 96.5  (96.3 – 96.7) |
| Testing | 0.976  (0.975 – 0.976) | 87.5  (87.0 – 87.9) | 96.7  (96.5 – 96.9) | 91.6  (91.1 – 92.1) | 95.0  (94.8 – 95.1) | 95.8  (95.7 – 96.0) | 94.0  (93.8 – 94.3) |

AUC, area under receiver operating characteristic; CI, confidence interval; NPV, negative predictive value; PPV, positive predictive value
