# Supplementary material for: Developing a machine-learning model for real-time prediction of successful extubation in mechanically ventilated patients using time-series ventilator-derived parameters
Source: Front Med (Lausanne). 2023 May 9;10:1167445. doi: 10.3389/fmed.2023.1167445 (PMC10203709; doi:10.3389/fmed.2023.1167445)

**Supplementary Figure S1.**

Flowchart for using time-series ventilator-derived parameters to develop a machine-learning model.


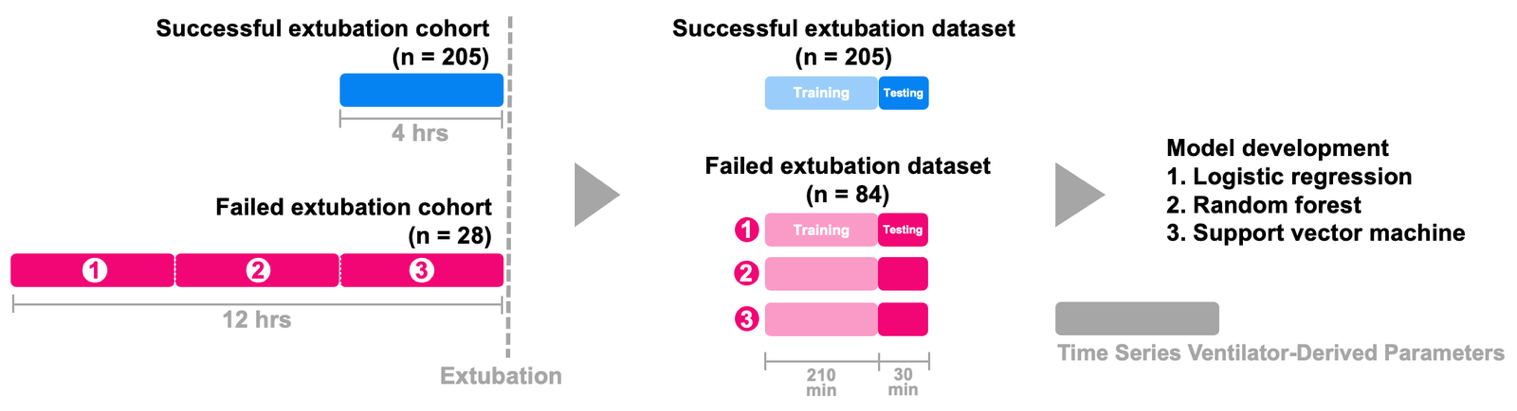


**Supplementary Figure S2.**

SHapley Additive exPlanations (SHAP) values of ventilator-derived features in the random forest (RF) model. (A) The summary plot of the SHAP value of all ventilator-derived features in the RF model. (B) An example of a successful extubation case with the SHAP value prediction result. (C) An example of an extubation failure case with the SHAP value prediction result. FiO_2_, fraction of inspiration oxygen; PEEP, positive end-expiratory pressure; Pmean, mean airway pressure; Ppeak, peak airway pressure; RR, respiratory rate; Vte, expiratory tidal volume.


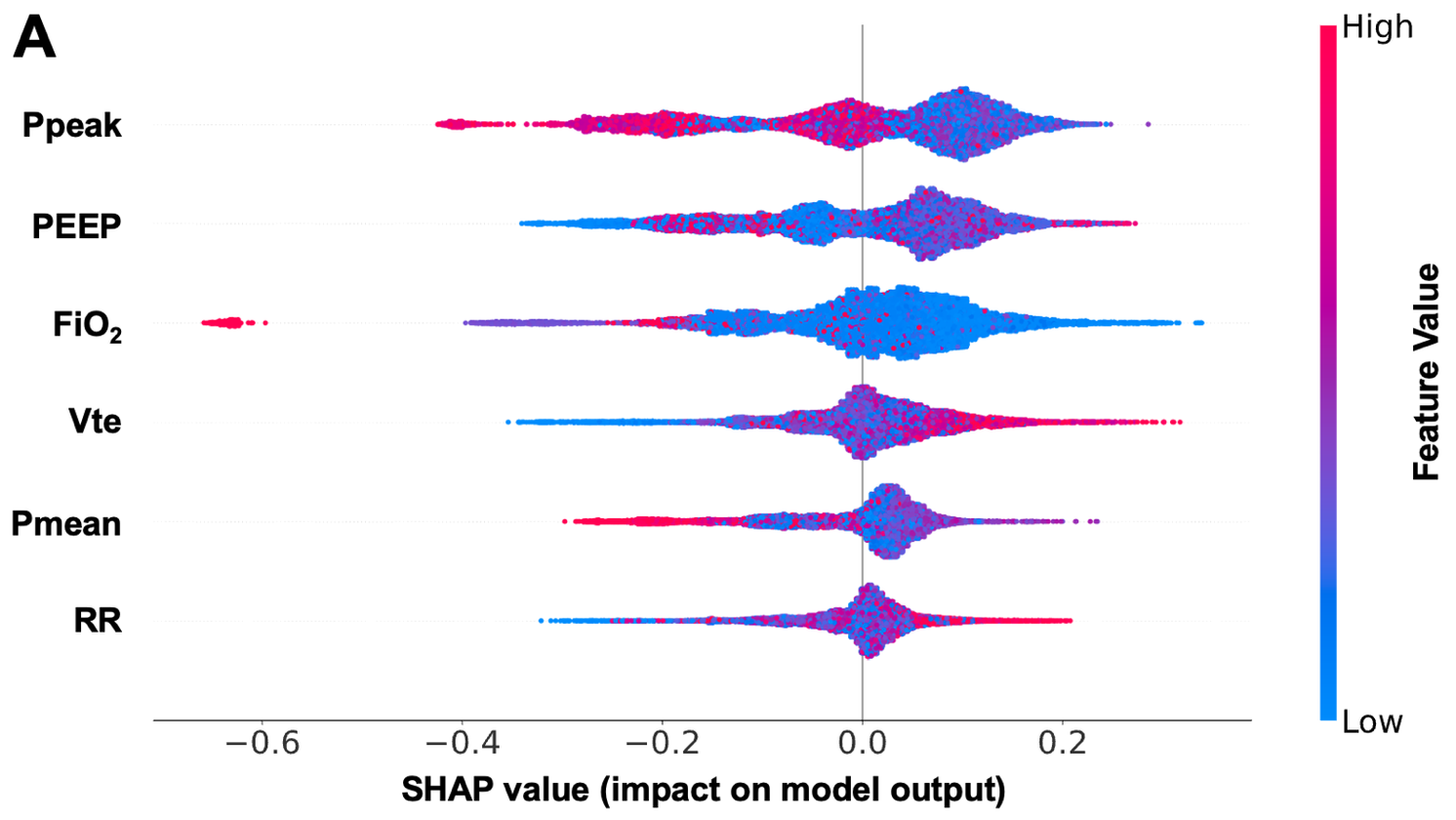


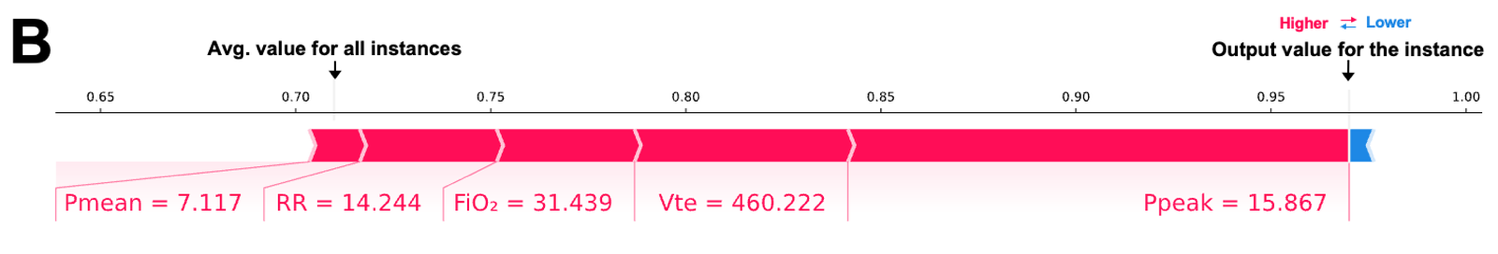


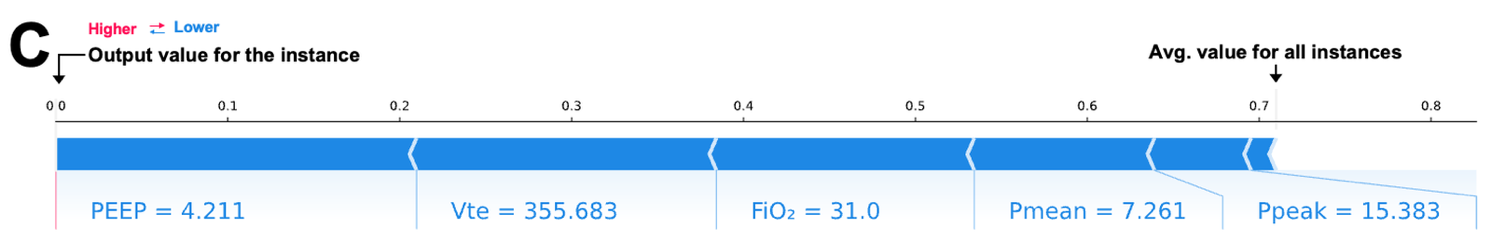


**Supplementary Figure S3.**

Distribution of probability density of predictive values by the random forest (RF) model for the testing dataset.


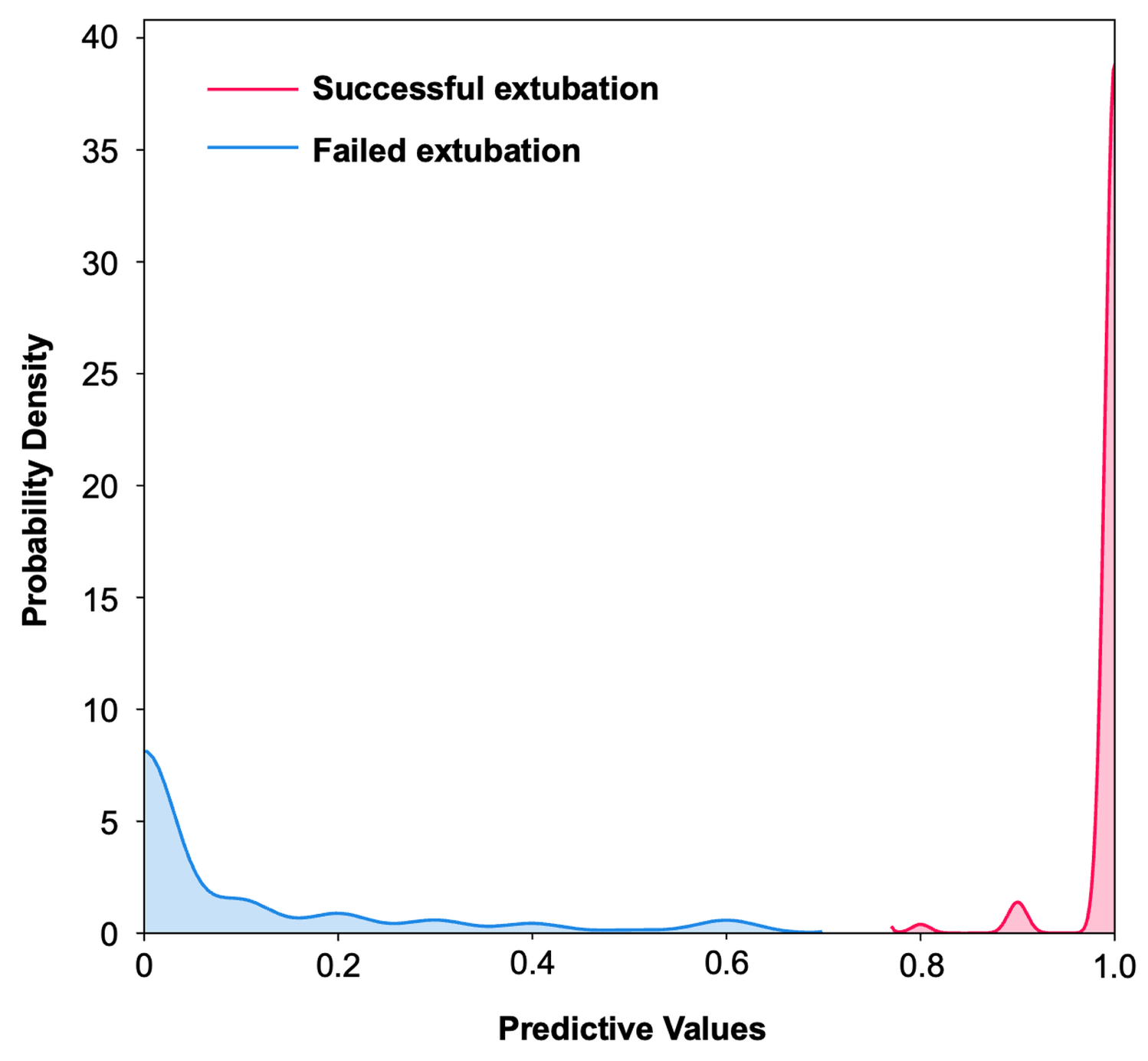

Supplement: Supplementary file 4 [file Data_Sheet_1.docx]
